# Clonal haematopoiesis is not prevalent in survivors of childhood cancer

**DOI:** 10.1111/bjh.14630

**Published:** 2017-04-03

**Authors:** Grace Collord, Naomi Park, Marina Podestà, Monica Dagnino, Daniela Cilloni, David Jones, Ignacio Varela, Francesco Frassoni, George S. Vassiliou

**Affiliations:** ^1^ Wellcome Trust Sanger Institute Cambridge UK; ^2^ Department of Paediatrics Cambridge University Hospitals NHS Foundation Trust Cambridge UK; ^3^ Laboratorio Cellule Staminali e Terapie Cellulari Istituto Giannina Gaslini IRCCS Genova Italy; ^4^ Department of Clinical and Biological Sciences University of Turin Turin Italy; ^5^ Instituto de Biomedicina y Biotecnología de Cantabria Cantabria Spain; ^6^ Department of Haematology University of Cambridge Cambridge UK; ^7^ Department of Haematology Cambridge University Hospitals NHS Foundation Trust Cambridge UK

**Keywords:** haematopoiesis, late effects of therapy, haematopoietic stem cells, paediatric cancer, clonal evolution

Clonal haematopoiesis driven by leukaemia‐associated somatic mutations is a common feature of ageing (Link & Walter, 2016). This phenomenon, termed clonal haematopoiesis of indeterminate potential (CHIP), is associated with an increased risk of haematological malignancies and all‐cause mortality (Link & Walter, 2016). Recent empirical evidence and computational models suggest that mutation acquisition may not be the major rate‐limiting factor in the emergence of CHIP (Altrock *et al*, 2015; McKerrell *et al*, 2015; Link & Walter, 2016; Young *et al*, 2016). Instead, clonal expansion of mutant haematopoietic stem cells (HSCs) probably reflects the interaction between the effects of driver mutations and selection pressures prevailing in the bone marrow microenvironment (Link & Walter, 2016). Notably, cytotoxic therapies have been shown to favour expansion of pre‐malignant haematopoietic clones (Link & Walter, 2016). Furthermore, both adult and paediatric cancer patients treated with intensive chemoradiotherapy display an earlier onset of ageing‐associated morbidities and an elevated risk of therapy‐related myeloid neoplasms (t‐MN) and other secondary malignancies (Rowland & Bellizzi, 2014). A recent study in adult cancer patients found that CHIP was more prevalent than in the general population and was strongly associated with t‐MN and overall mortality (Gibson *et al*, 2017). Although CHIP is extremely rare in healthy young individuals, its prevalence and prognostic significance in paediatric cancer patients has not been studied. We therefore performed targeted deep sequencing of peripheral blood DNA from 84 childhood cancer survivors to search for subclonal mutations common in t‐MN and adult clonal haematopoiesis. No individuals with somatic variants at these loci were identified. Whilst our findings could be explained by a rarity of driver mutations, the fact that human HSCs accrue somatic variants from the first decade of life (Welch *et al*, 2012) proposes the alternative possibility that such mutations may not confer clonal advantage in the young.

We obtained peripheral blood DNA samples from patients enrolled on long‐term follow‐up after treatment for a paediatric malignancy and from three age‐matched controls with no cancer history. Written informed consent was obtained for sample collection and DNA sequencing from all patients or their guardian in accordance with the Declaration of Helsinki and protocols approved by the relevant institutional ethics committees (approval numbers 09REG2015, 1‐09/12/2015). The median age at cancer diagnosis was 4·5 years, and the commonest malignancies were acute lymphoblastic leukaemia (*n* = 21), neuroblastoma (*n* = 17) and non‐Hodgkin lymphoma (*n* = 10). Nineteen patients had received a HSC transplant (8 allogeneic and 11 autologous). The median interval between completion of cancer treatment and blood sampling was 6 years (range 2–25). Patient characteristics are summarized in Table SI.

We performed targeted next generation sequencing (NGS) using multiplex polymerase chain reaction to amplify 32 regions of 14 genes frequently mutated in CHIP or t‐MN (Table 1) (McKerrell *et al*, 2015; Link & Walter, 2016; Gibson *et al*, 2017). For this we extended a previously validated assay that detected clonal haemopoiesis in 2·6% of unselected adults (McKerrell *et al*, 2015), to include all coding exons of *TP53* and *PPM1D*, genes implicated in t‐MN pathogenesis (Rowland & Bellizzi, 2014; Link & Walter, 2016; Gibson *et al*, 2017). Primer design and sequencing was performed as described previously (McKerrell *et al*, 2015); see Table SII for primer sequences. Reads were aligned to human genome build GRCh37 using the Burrows‐Wheeler Aligner (Li & Durbin, 2010) and analysed for somatic single nucleotide variants. Allele counts were generated using an in‐house script (https://github.com/cancerit/alleleCount), considering only loci with ≥1000 reads and minimum base and mapping quality of 25 and 35, respectively. Somatic mutations with variant allele frequency (VAF) ≥0·008 (McKerrell *et al*, 2015) were sought and examined visually and by interrogation with the Shearwater algorithm (https://github.com/mg14/deepSNV) (Gerstung *et al*, 2014).

**Table 1:** Genomic regions sequenced

Gene	Chromosome	Target codon/exon
*NRAS*	1	p.G12
*SF3B1*	2	p.K666; p.K700
*DNMT3A*	2	p.R882
*IDH1*	2	p.R132
*KIT*	4	exon 17
*NPM1*	5	exon 12
*JAK2*	9	p.V617
*KRAS*	12	p.G12
*IDH2*	15	p.R140; p.R172
*PPM1D*	17	exons 1–6
*TP53*	17	exons 1–12
*SRSF2*	17	p.P95
*ASXL1*	20	exon 12
*U2AF1*	21	p.S34; p.Q157

The median sequencing depth across regions of interest was 5·3 × 10^3^. No somatic mutations with VAF ≥ 0·008 were observed in any of our patients or controls, demonstrating that CHIP driven by mutations at these loci is not prevalent in young individuals who have received cytotoxic treatment. By contrast, Gibson *et al* (2017) identified post‐chemotherapy CHIP (VAF > 0·02) in 29·9% of 401 adult lymphoma patients. Notably, mutations in *PPM1D*, a regulator of *TP53*, were the commonest CHIP drivers (Gibson *et al*, 2017). Similarly, several smaller studies have demonstrated clonal expansion in older patients undergoing chemoradiotherapy for other cancers (Link & Walter, 2016). An investigation of haematopoietic clonal dynamics in 15 adult acute myeloid leukaemia patients found that, after induction chemotherapy, five had marked expansion of clones unrelated to their leukaemia (Link & Walter, 2016). Most clones carried canonical leukaemia mutations and continued to expand years after remission (Link & Walter, 2016). In a study exploring the clonal origins of t‐MN, *TP53*‐mutated clones expanded dramatically after cytotoxic treatment, whereas the same mutations demonstrated very modest clonal advantage in healthy individuals (Link & Walter, 2016). In light of the above, our findings have two plausible explanations: (i) that somatic driver mutations are very uncommon in young individuals even after exposure to chemotherapy or (ii) that accrual of such mutations is insufficient to trigger clonal expansion in this age group. The latter is supported by findings that oncogenic mutations begin accumulating early in life (Welch *et al*, 2012) and that cancer‐associated mutations are less able to drive clonal expansion in young compared to old stem cells (Zhu *et al*, 2016). The fact that bona‐fide driver mutations do not always lead to haematopoietic clonal expansion, even after several years, was highlighted by Young *et al* (2016), using ultra‐sensitive sequencing. Therefore our results should not be taken to reflect absence of potentially oncogenic HSC mutations in young cancer survivors. Rather, it is possible that even canonical leukaemogenic mutations may not commonly drive clonal outgrowth in children and young adults despite exposure to extreme haematopoietic stress, implicating age‐related changes in HSCs and/or their microenvironment as key determinants of relative fitness. More sensitive DNA sequencing methods may enable detection of very rare cells harbouring known CHIP drivers mutations in similar patient cohorts, which would lend support to this hypothesis. Studies of larger numbers of paediatric cancer survivors are needed to identify rare individuals with CHIP after chemoradiotherapy, whose particular characteristics may offer insights into factors facilitating clonal outgrowth of mutated HSCs. Furthermore, in view of the shifting patterns of mutations driving CHIP in different adult age groups (McKerrell *et al*, 2015), selective pressures particular to a less mature bone marrow environment may confer clonal advantage on a distinct spectrum of somatic variants in the very young. Although a much broader screening approach is required to identify such mutations, the potential role for CHIP as a biomarker for patient risk‐stratification (Gibson *et al*, 2017) may render this a worthwhile endeavour.

## Author contributions

GSV, GC and FF conceived and designed the study. NH designed sequencing assays. GC performed experiments and bioinformatics analysis. GC and GSV wrote the manuscript with input from FF. DJ and IV wrote scripts and contributed to analysis strategy. FF, MP, MD and DC contributed to sample acquisition and patient recruitment.

## Supporting information


**Table SI.** Patient characteristics
**Table SII.** Primer sequences
