# Supplementary material for: Clonal haematopoiesis is not prevalent in survivors of childhood cancer
Source: Br J Haematol. 2017 Apr 3;181(4):537–9. doi: 10.1111/bjh.14630 (PMC6001539; doi:10.1111/bjh.14630)
Supplement: Supplementary file 1 — Table SI. Patient characteristics Table SII. Primer sequences [file BJH-181-537-s001.pdf]

**Table SI | Patient Characteristics**

| Study ID | Sex | Diagnosis | Age at diagnosis | Months since cytotoxic treatment |
|---|---|---|---|---|
| **1** | F | NB | 15.4 | 64.3 |
| **2** | M | RMS | 11.1 | 21.7 |
| **3** | F | ALL | 5.7 | 132.4 |
| **4** | NA | ALL | NA | NA |
| **5*** | F | ALL | 1.1 | 106.4 |
| **6** | F | ALL | 6.1 | 80.3 |
| **7** | M | NB | 6.3 | 231.9 |
| **8§** | M | NHL | 4.7 | 176.2 |
| **9** | f | ALL | 1.7 | 52.6 |
| **10§** | M | ALL | 6.9 | 298.2 |
| **11** | F | GCT | 9.3 | 25.9 |
| **12** | M | RMS | 6.0 | 102.9 |
| **13** | F | NHL | 7.1 | 103.9 |
| **14** | M | ALL | 6.9 | 177.4 |
| **15** | F | NHL | 9.4 | 80.1 |
| **16** | M | NB | 0.6 | 94.0 |
| **17*** | M | LL | 5.8 | 55.4 |
| **18** | M | HL | 14.8 | 136.6 |
| **19** | M | WT | 0.8 | 57.3 |
| **20** | M | RMS | 3.1 | 47.6 |
| **21** | F | ALL | 9.1 | 35.7 |
| **22** | M | HL | 10.9 | 43.5 |
| **23** | M | ALL | 4.0 | 49.5 |
| **24** | M | HL | 14.2 | 42.5 |
| **25** | M | HB | 0.3 | 112.9 |
| **26*** | M | ALL | 0.6 | 81.1 |
| **27** | M | HL | 7.1 | 86.2 |
| **28** | M | GCT | 15.4 | 26.7 |
| **29** | M | RMS | 5.8 | 76.2 |
| **30§** | M | NHL | 15.5 | 46.6 |
| **31** | M | HL | 25.4 | 48.5 |
| **32§** | M | ESFT | 4.6 | 141.5 |
| **33** | M | LL | 9.3 | 112.9 |
| **34** | F | ESFT | 3.3 | 74.3 |
| **35*** | M | NB | 2.3 | 102.9 |
| **36** | M | NHL | 2.0 | 46.4 |
| **37** | M | NB | 3.4 | 166.4 |
| **38** | F | NB | 1.7 | 124.8 |
| **39*** | M | LL | 3.2 | 112.9 |
| **40§** | M | NB | 0.5 | 289.3 |
| **41** | F | WT | 3.1 | 105.9 |
| **42** | M | NB | 0.9 | 268.4 |
| **43** | F | NB | 0.6 | 238.8 |
| **44** | M | NHL | 5.8 | 183.2 |

| | | | | |
|---|---|---|---|---|
| **45** | M | RMS | 8.4 | 192.2 |
| **46** | M | NRSTS | 4.3 | 105.9 |
| **47** | M | ALL | 3.0 | 58.3 |
| **48** | M | ALL | 3.9 | 35.7 |
| **49** | M | NB | 5.5 | NA |
| **50** | F | ESFT | 13.4 | 69.2 |
| **51*** | M | ALL | 4.7 | 89.1 |
| **52** | F | CCA | 12.8 | 41.5 |
| **53** | M | NB | 4.0 | 73.3 |
| **54** | F | WT | 4.8 | 63.4 |
| **55** | M | HL | 15.3 | 46.4 |
| **56*** | M | ALL | 1.5 | 44.5 |
| **57** | M | NPC | 15.9 | 35.4 |
| **58** | F | NHL | 8.7 | 25.7 |
| **59** | M | ALL | 4.5 | 59.4 |
| **60** | M | ALL | 3.6 | 35.9 |
| **61** | M | NB | 5.8 | 34.5 |
| **62** | M | NHL | 2.6 | 59.3 |
| **63** | M | NHL | 9.1 | 62.4 |
| **64** | F | RMS | 3.0 | 80.1 |
| **65** | F | NB | 0.3 | 138.6 |
| **66** | F | RMS | 1.1 | 45.4 |
| **67** | F | ALL | 2.4 | 54.4 |
| **68** | M | NHL | 3.7 | 212.9 |
| **69** | F | NRSTS | 11.0 | 38.1 |
| **70** | M | NB | 0.4 | 45.4 |
| **71** | F | LCH | 3.7 | 88.1 |
| **72*** | F | LCH | 3.1 | 69.2 |
| **73** | F | WT | 3.8 | 142.7 |
| **74** | F | GCT | 0.0 | 131.8 |
| **75** | M | GCT | 15.4 | NA |
| **76§** | F | WT | 4.9 | 96.1 |
| **77** | F | ALL | 8.2 | 45.4 |
| **78§** | F | NB | 1.1 | 39.6 |
| **79** | M | ALL | 4.5 | 77.2 |
| **80§** | M | NB | 1.3 | 194.1 |
| **81** | M | ALL | 3.3 | 48.5 |
| **82** | F | NB | 0.3 | 75.2 |
| **83** | M | ALL | 3.0 | 75.2 |
| **84** | M | ESFT | 10.7 | 100.0 |

RMS, rhabdomyosarcoma; ALL, acute lympoblastic leukaemia; NB, neuroblastoma; NHL, non-Hodgkin lymphoma; GCT, germ cell tumour; LL, lymphoblastic lymphoma; HL, Hodgkin lymphoma; WT, Wilm's tumour; ESFT, Ewing sarcoma; NRSTS, non-rhabdomyosarcoma soft tissue sarcoma; NPC, nasopharyngeal sarcoma; CCA, choriocarcinoma; LCH, Langerhans cell histiocytosis; NA, no data. Patients who received a haematopoietic stem cell transplant (HSCT) are indicated with the symbols * (allogeneic HSCT) or § (autologous HSCT).

**Table SII | Primer Sequences**

| PLEX | PRIMER NAME | GENE | TARGETED EXON/CODON | PRIMER SEQUENCE3 |
|---|---|---|---|---|
| **1** | ASXL1_exon12_a_F | ASXL1 | exon12 | ACACTCTTTCCCTACACGACGCTCTTCCGAT CTGGACCCTCGCAGACATTAmAA |
| **1** | ASXL1_exon12_a_R | ASXL1 | exon12 | TCGGCATTCCTGCTGAACCGCTCTTCCGATC TGCTGTAGATCTGACGTACACmUT |
| **1** | ASXL1_exon12_b_F | ASXL1 | exon12 | ACACTCTTTCCCTACACGACGCTCTTCCGAT CTCAGTGGTGATGGTGGTGmAG |
| **1** | ASXL1_exon12_b_R | ASXL1 | exon12 | TCGGCATTCCTGCTGAACCGCTCTTCCGATC TGGCATCTCCTAGCCCATmCT |
| **1** | ASXL1_exon12_c_F | ASXL1 | exon12 | ACACTCTTTCCCTACACGACGCTCTTCCGAT CTCTACTACAGAGGGCTACAGTmUG |
| **1** | ASXL1_exon12_c_R | ASXL1 | exon12 | TCGGCATTCCTGCTGAACCGCTCTTCCGATC TCTTGCTCCTCATCATCACTTmUC |
| **1** | DNMT3A_p.R693C_F | DNMT3A | p.R693C | ACACTCTTTCCCTACACGACGCTCTTCCGAT CTCCTCATGTTCTTGGTGTTTTAT |
| **1** | DNMT3A_p.R693C_R | DNMT3A | p.R693C | TCGGCATTCCTGCTGAACCGCTCTTCCGATC TTTTTCTCCCCCAGGGTATTTG |
| **1** | IDH1_p.R132H_F | IDH1 | p.R132H | ACACTCTTTCCCTACACGACGCTCTTCCGAT CTTAAATGTGTGTAAATATACAGTTAT |
| **1** | IDH1_p.R132H_R | IDH1 | p.R132H | TCGGCATTCCTGCTGAACCGCTCTTCCGATC TATTATCTGCAAAAATATCCCCC |
| **1** | IDH2_p.R172K_IDH2_p.R140Q_F | IDH2 | p.R172K, p.R140Q | ACACTCTTTCCCTACACGACGCTCTTCCGAT CTAAGAGGATGGCTAGGCGAGGA |
| **1** | IDH2_p.R172K_IDH2_p.R140Q_R | IDH2 | p.R172K, p.R140Q | TCGGCATTCCTGCTGAACCGCTCTTCCGATC TCTCACAGAGTTCAAGCTGAAG |
| **1** | JAK2_p.V617F_F | JAK2 | p.V617F | ACACTCTTTCCCTACACGACGCTCTTCCGAT CTAGTCTTTCTTTGAAGCAGCAAG |
| **1** | JAK2_p.V617F_R | JAK2 | p.V617F | TCGGCATTCCTGCTGAACCGCTCTTCCGATC TAGTTTACACTGACACCTAGCTG |
| **1** | KIT_exon17_F | KIT | exon17 | ACACTCTTTCCCTACACGACGCTCTTCCGAT CTTGGTTTTCTTTTCTCCTCCAAC |
| **1** | KIT_exon17_R | KIT | exon17 | TCGGCATTCCTGCTGAACCGCTCTTCCGATC TTCCTTTGCAGGACTGTCAAG |
| **1** | KRAS_p.G12R_F | KRAS | p.G12R | ACACTCTTTCCCTACACGACGCTCTTCCGAT CTTGTTGGATCATATTCGTCCACA |
| **1** | KRAS_p.G12R_R | KRAS | p.G12R | TCGGCATTCCTGCTGAACCGCTCTTCCGATC TAAGGTACTGGTGGAGTATTTGA |
| **1** | NPM1_p.L287fs*13_F | NPM1 | p.L287fs*13 | ACACTCTTTCCCTACACGACGCTCTTCCGAT CTTGTTTGGAATTAAATTACATCTGA |
| **1** | NPM1_p.L287fs*13_R | NPM1 | p.L287fs*13 | TCGGCATTCCTGCTGAACCGCTCTTCCGATC TAAAATTTTTTAACAAATTGTTTAAACT |
| **1** | NRAS_p.G12D_F | NRAS | p.G12D | ACACTCTTTCCCTACACGACGCTCTTCCGAT CTATGGGTAAAGATGATCCGACAA |
| **1** | NRAS_p.G12D_R | NRAS | p.G12D | TCGGCATTCCTGCTGAACCGCTCTTCCGATC TCGCCAATTAACCCTGATTACTG |
| **1** | SF3B1_p.K666N_F | SF3B1 | p.K666N | ACACTCTTTCCCTACACGACGCTCTTCCGAT CTACCCTGTCTCCTAAAGAAAAAA |
| **1** | SF3B1_p.K666N_R | SF3B1 | p.K666N | TCGGCATTCCTGCTGAACCGCTCTTCCGATC TTAGAGCTTTTGCTGTTGTAGC |
| **1** | SF3B1_p.K700E_F | SF3B1 | p.K700E | ACACTCTTTCCCTACACGACGCTCTTCCGAT CTTAGTAATTTAGATTTATGTCGCC |
| **1** | SF3B1_p.K700E_R | SF3B1 | p.K700E | TCGGCATTCCTGCTGAACCGCTCTTCCGATC |

| | | | | |
|---|---|---|---|---|
| | | | | TGGCATAGTTAAAACCTGTGTTT |
| **1** | SRSF2_p.P95L_F | SRSF2 | p.P95L | ACACTCTTTCCCTACACGACGCTCTTCCGAT CTTGCTTCGCCGCGGACCTTTGT |
| **1** | SRSF2_p.P95L_R | SRSF2 | p.P95L | TCGGCATTCCTGCTGAACCGCTCTTCCGATC TGAGGACGCTATGGATGCCATG |
| **1** | U2AF1_p.Q157R_F | U2AF1 | p.Q157R | ACACTCTTTCCCTACACGACGCTCTTCCGAT CTGGGTTGGAAGGAGACATTTAmCT |
| **1** | U2AF1_p.Q157R_R | U2AF1 | p.Q157R | TCGGCATTCCTGCTGAACCGCTCTTCCGATC TGAAAAGGCTGTGATTGACTTmGA |
| **1** | U2AF1_p.S34F_F | U2AF1 | p.S34F | ACACTCTTTCCCTACACGACGCTCTTCCGAT CTCGATCACCTGCCTCACTATTmAT |
| **1** | U2AF1_p.S34F_R | U2AF1 | p.S34F | TCGGCATTCCTGCTGAACCGCTCTTCCGATC TTTTCAAAATTGGAGCATGTCmGT |
| **2** | PPM1D_exon1_a_F | PPM1D | exon1 | ACACTCTTTCCCTACACGACGCTCTTCCGAT CTGAGCGCCTAGTGTGTCmUC |
| **2** | PPM1D_exon1_a_R | PPM1D | exon1 | TCGGCATTCCTGCTGAACCGCTCTTCCGATC TGCCTTTCCCCGAGACTmUC |
| **2** | PPM1D_exon1_c_F | PPM1D | exon1 | ACACTCTTTCCCTACACGACGCTCTTCCGAT CTGTTCCTCCGTGGCCTTmUT |
| **2** | PPM1D_exon1_c_R | PPM1D | exon1 | TCGGCATTCCTGCTGAACCGCTCTTCCGATC TCAAACAAGCCAGGGAACTTmAC |
| **2** | PPM1D_exon3_F | PPM1D | exon3 | ACACTCTTTCCCTACACGACGCTCTTCCGAT CTACTGAGCTATCTTAGTTGTTmGT |
| **2** | PPM1D_exon3_R | PPM1D | exon3 | TCGGCATTCCTGCTGAACCGCTCTTCCGATC TTGCCAAGTAAGGGTTTAGTTmCT |
| **2** | PPM1D_exon5_a_F | PPM1D | exon5 | ACACTCTTTCCCTACACGACGCTCTTCCGAT CTACAGATGTAGTGGCAGCTAAmAT |
| **2** | PPM1D_exon5_a_R | PPM1D | exon5 | TCGGCATTCCTGCTGAACCGCTCTTCCGATC TGTCATCACACAGGTTTCTTGmAC |
| **2** | PPM1D_exon6_a_F | PPM1D | exon6 | ACACTCTTTCCCTACACGACGCTCTTCCGAT CTTGCATAGATTTGTTGAGTTCTmGG |
| **2** | PPM1D_exon6_a_R | PPM1D | exon6 | TCGGCATTCCTGCTGAACCGCTCTTCCGATC TTGGAAGGCTATTATTCAAAGAATmCA |
| **2** | PPM1D_exon6_c_F | PPM1D | exon6 | ACACTCTTTCCCTACACGACGCTCTTCCGAT CTTTAGAAGAGTCCAATTCTGGmCC |
| **2** | PPM1D_exon6_c_R | PPM1D | exon6 | TCGGCATTCCTGCTGAACCGCTCTTCCGATC TTCAACATCGGCACCAAATTTmAA |
| **2** | TP53_exon1_F | TP53 | exon1 | ACACTCTTTCCCTACACGACGCTCTTCCGAT CTTTCAAAGACCCAAAACCCAAmAA |
| **2** | TP53_exon1_R | TP53 | exon1 | TCGGCATTCCTGCTGAACCGCTCTTCCGATC TTTGATTTGAATTCCCGTTGTmCC |
| **2** | TP53_exon10_a_F | TP53 | exon10 | ACACTCTTTCCCTACACGACGCTCTTCCGAT CTATTGAAGTCTCATGGAAGCCmAG |
| **2** | TP53_exon10_a_R | TP53 | exon10 | TCGGCATTCCTGCTGAACCGCTCTTCCGATC TCGGACGATATTGAACAATGGmUT |
| **2** | TP53_exon10_b_F | TP53 | exon10 | ACACTCTTTCCCTACACGACGCTCTTCCGAT CTGAAGGGACAGAAGATGACAmGG |
| **2** | TP53_exon10_b_R | TP53 | exon10 | TCGGCATTCCTGCTGAACCGCTCTTCCGATC TGACTGCTCTTTTCACCCATCmUA |
| **2** | TP53_exon11_F | TP53 | exon11 | ACACTCTTTCCCTACACGACGCTCTTCCGAT CTGGACTGTAGATGGGTGAAAAmGA |
| **2** | TP53_exon11_R | TP53 | exon11 | TCGGCATTCCTGCTGAACCGCTCTTCCGATC TAGACCTATGGAAACTGTGAGmUG |
| **2** | TP53_exon12_F | TP53 | exon12 | ACACTCTTTCCCTACACGACGCTCTTCCGAT CTAACGTTGTTTTCAGGAAGTCmUG |

| | | | | |
|---|---|---|---|---|
| **2** | TP53_exon2_F | TP53 | exon2 | ACACTCTTTCCCTACACGACGCTCTTCCGATCTTGAGAATGGAATCCTATGGCmUT |
| **2** | TP53_exon2_R | TP53 | exon2 | TCGGCATTCCTGCTGAACCGCTCTTCCGATCTCATGTTGCTTTTGTACCGTCmAT |
| **2** | TP53_exon3_F | TP53 | exon3 | ACACTCTTTCCCTACACGACGCTCTTCCGATCTGGCTAGGCTAAGCTATGATGmUT |
| **2** | TP53_exon3_R | TP53 | exon3 | TCGGCATTCCTGCTGAACCGCTCTTCCGATCTGCTCCTGGTTGTAGCTAACTmAA |
| **2** | TP53_exon5_F | TP53 | exon5 | ACACTCTTTCCCTACACGACGCTCTTCCGATCTTTTCCACTTGATAAGAGGTCmCC |
| **2** | TP53_exon5_R | TP53 | exon5 | TCGGCATTCCTGCTGAACCGCTCTTCCGATCTGAAGAGAATCTCCGCAAGAAmAG |
| **2** | TP53_exon7_F | TP53 | exon7 | ACACTCTTTCCCTACACGACGCTCTTCCGATCTGAGAGGTGGATGGGTAGTAGmUA |
| **2** | TP53_exon7_R | TP53 | exon7 | TCGGCATTCCTGCTGAACCGCTCTTCCGATCTATCTTGGGCCTGTGTTATCmUC |
| **2** | TP53_exon9_F | TP53 | exon9 | ACACTCTTTCCCTACACGACGCTCTTCCGATCTAATCAGTGAGGAATCAGAGmGC |
| **2** | TP53_exon9_R | TP53 | exon9 | TCGGCATTCCTGCTGAACCGCTCTTCCGATCTTTTCAACTCTGTCTCCTTCCmUC |
| **3** | PPM1D_exon1_b_F | PPM1D | exon1 | ACACTCTTTCCCTACACGACGCTCTTCCGATCTAACCGACGGCTGAAGAAmAA |
| **3** | PPM1D_exon1_b_R | PPM1D | exon1 | TCGGCATTCCTGCTGAACCGCTCTTCCGATCTCTTCTTGATGAAACCCCACAmAG |
| **3** | PPM1D_exon2_F | PPM1D | exon2 | ACACTCTTTCCCTACACGACGCTCTTCCGATCTACTTGCAAGAGTGAAATATTmUT |
| **3** | PPM1D_exon2_R | PPM1D | exon2 | TCGGCATTCCTGCTGAACCGCTCTTCCGATCTGAAAGAGAAAACGACAGAATmGT |
| **3** | PPM1D_exon4_F | PPM1D | exon4 | ACACTCTTTCCCTACACGACGCTCTTCCGATCTGCTTCCAACTAATACTTCTTGmCT |
| **3** | PPM1D_exon4_R | PPM1D | exon4 | TCGGCATTCCTGCTGAACCGCTCTTCCGATCTACCAAAACAATGTTTAGACAmAC |
| **3** | PPM1D_exon5_b_F | PPM1D | exon5 | ACACTCTTTCCCTACACGACGCTCTTCCGATCTAGTGCCATAGTAATCTGCATmCT |
| **3** | PPM1D_exon5_b_R | PPM1D | exon5 | TCGGCATTCCTGCTGAACCGCTCTTCCGATCTTCGAGTTCAAATCCAAAATCCmUG |
| **3** | PPM1D_exon6_b_F | PPM1D | exon6 | ACACTCTTTCCCTACACGACGCTCTTCCGATCTTACCCTCAAAAGATCCAGAAmCC |
| **3** | PPM1D_exon6_b_R | PPM1D | exon6 | TCGGCATTCCTGCTGAACCGCTCTTCCGATCTTCGACTTAAGCCATTTCGTCmUA |
| **3** | TP53_exon12_R | TP53 | exon12 | TCGGCATTCCTGCTGAACCGCTCTTCCGATCTGGATCCCCACTTTTCCTCTmUG |
| **3** | TP53_exon4_F | TP53 | exon4 | ACACTCTTTCCCTACACGACGCTCTTCCGATCTTCAGGCAAAGTCATAGAACCmAT |
| **3** | TP53_exon4_R | TP53 | exon4 | TCGGCATTCCTGCTGAACCGCTCTTCCGATCTTGACTGTTTTACCTGCAATTmGG |
| **3** | TP53_exon6_F | TP53 | exon6 | ACACTCTTTCCCTACACGACGCTCTTCCGATCTAGAGGCAAGGAAAGGTGATAmAA |
| **3** | TP53_exon6_R | TP53 | exon6 | TCGGCATTCCTGCTGAACCGCTCTTCCGATCTTAGGACCTGATTTCCTTACTmGC |
| **3** | TP53_exon8_F | TP53 | exon8 | ACACTCTTTCCCTACACGACGCTCTTCCGATCTTTGCACATCTCATGGGGTTAmUA |
| **3** | TP53_exon8_R | TP53 | exon8 | TCGGCATTCCTGCTGAACCGCTCTTCCGATCTTGATTCCTCACTGATTGCTCmUT |

Nucleotide sequences for multiplexed primers used in plexes 1 - 3.
* Consecutive primers constitute forward (F) and reverse (R) primer pairs for the indicated loci

† Forward primers format: 5’ ACACTCTTTCCCTACACGACGCTCTTCCGATCT-[gene-specific forward] 3’,
Reverse primerformat:5’ TCGGCATTCCTGCTGAACCGCTCTTCCGATCT-[gene-specific reverse] 3’

‡ "m" denotes a single 2'-O-Methyl base in place of the DNA base, used in order to minimise potential primer dimers
